# Subcongenic analysis of a quantitative trait locus affecting body weight and glucose metabolism in *zinc transporter 7* (*znt7)*-knockout mice

**DOI:** 10.1186/s12863-019-0715-2

**Published:** 2019-02-18

**Authors:** L. Huang, S. Tepaamorndech, C. P. Kirschke, Y. Cai, J. Zhao, Xiaohan Cao, Andrew Rao

**Affiliations:** 1 Obesity and Metabolism Research Unit, USDA/ARS/Western Human Nutrition Research Center, 430 West Health Sciences Drive, Davis, CA 95616 USA; 2 Integrative Genetics and Genomics Graduate Group, University of California Davis, One Shields Avenue, Davis, CA 95616 USA; 3 Present Address: Food Biotechnology Research Unit, National Center for Genetic Engineering and Biotechnology (BIOTEC), 113 Thailand Science Park, Phahonyothin Road, Pathum Thani, 12120 Thailand; 4 Graduate Group of Nutritional Biology, University of California Davis, One Shields Avenue, Davis, CA 95616 USA; 5 Department of Nutrition, University of California Davis, One Shields Avenue, Davis, CA 95616 USA; 6 School of Food Science, Nanjing Xiaozhuang University, Nanjing, 211171 Jiangsu China; 7 Food Science and Technology, University of California Davis, One Shields Avenue, Davis, CA 95616 USA

**Keywords:** Slc30a7, ZnT7, QTL, Congenic mice, Zinc transporter, Body weight, Glucose metabolism, Htatip2

## Abstract

**Background:**

A genome-wide mapping study using male F_2_ *zinc transporter 7*-knockout mice (*znt7*-KO) and their wild type littermates in a mixed 129P1/ReJ (129P1) and C57BL/6J (B6) background identified a quantitative trait locus (QTL) on chromosome 7, which had a synergistic effect on body weight gain and fat deposit with the *znt7*-null mutation.

**Results:**

The genetic segment for body weight on mouse chromosome 7 was investigated by newly created subcongenic *znt7*-KO mouse strains carrying different lengths of genomic segments of chromosome 7 from the 129P1 donor strain in the B6 background. We mapped the sub-QTL for body weight in the proximal region of the previously mapped QTL, ranging from 47.4 to 64.4 megabases (Mb) on chromosome 7. The 129P1 donor allele conferred lower body weight gain and better glucose handling during intraperitoneal glucose challenge than the B6 allele control. We identified four candidate genes, including *Htatip2*, *E030018B13Rik*, *Nipa1*, and *Atp10a*, in this sub-QTL using quantitative RT-PCR and cSNP detection (single nucleotide polymorphisms in the protein coding region).

**Conclusions:**

This study dissected the genetic determinates of body weight and glucose metabolism in *znt7*-KO mice. The study demonstrated that a 17-Mb long 129P1 genomic region on mouse chromosome 7 conferred weight reduction and improved glucose tolerance in *znt7*-KO male mice. Among the four candidate genes identified, *Htatip2* is the most likely candidate gene involved in the control of body weight based on its function in regulation of lipid metabolism. The candidate genes discovered in this study lay a foundation for future studies of their roles in development of metabolic diseases, such as obesity and type 2 diabetes.

**Electronic supplementary material:**

The online version of this article (10.1186/s12863-019-0715-2) contains supplementary material, which is available to authorized users.

## Background

Cellular zinc homeostasis is largely maintained by two families of zinc transporters, Slc30a (Slc30a1–10 or ZnT1–10) [1] and Slc39a (Slc39a1–14 or Zip1–14) [2]. ZnT and Zip proteins act in concert to mobilize zinc across the cytoplasmic or organelle membrane to maintain free zinc under tight control in a given cell. [1]. ZnT and Zip transporters move zinc in opposite directions i.e., ZnT proteins sequester zinc into intracellular compartments or transport zinc out to the extracellular space when cellular zinc is abundant while Zip proteins do the opposite during zinc depletion [1, 2]. The expression of zinc transporters exhibits both ubiquitous and tissue- or cell-specific patterns [1]. Among the 10 ZnT proteins, ZnT2–4, ZnT8, and ZnT10 are more narrowly expressed than the others [3–6]. ZnT1 is ubiquitously expressed functioning to export zinc out of the cell [7]. The expression of ZnT5–7 and ZnT9 is widespread with varying amounts depending on tissues or cell types [8–11].

ZnT7 is responsible for zinc accumulation in the Golgi apparatus of the cell [10, 12]. A null mutation in the mouse *znt7* gene results in reduced cellular zinc accumulation, which leads to mild zinc deficiency in mice [13]. *znt7*-knockout (*znt7*-KO) mice display decreased weight gain and fat deposit as well as reduced pancreatic insulin production and secretion. Moreover, male *znt7*-KO mice are insulin resistant in the congenic B6 genetic background [13–15], indicating that the effect of the *znt7*-null mutation on phenotypic expressivity is dependent on the genetic background of mouse strains [16]. As a result, male *znt7*-KO mice are highly susceptible to diet-induced glucose intolerance [14]. Zinc deficiency affecting lipid metabolism has been reported in humans and animal models [15, 17–21]. Zinc depletion reduces fatty acid synthesis from glucose in epididymal fat [22] whereas zinc supplementation stimulates lipid synthesis from glucose in adipocytes [23, 24]. A genome-wide quantitative trait locus (QTL) mapping study using 129P1/ReJ (129P1) and B6 mouse strains revealed that the degree of the effect of *znt7*-KO on weight and fat deposit was associated with a genomic region on mouse chromosome 7 [16]. This QTL ranges from 40.3 to 81.3 megabases (Mb) on chromosome 7 (based on the mouse genome GRCm38.p1 assembly) and confers reduced body weight and fat deposition in mice with a 129P1 donor allele in the B6 background [16].

In this study, we created two subcongenic mouse stains containing genomic segments from the 129P1 donor allele in the B6 recipient background to identify complex trait genes involved in body weight, fatness, and glucose metabolism. We used congenic *znt7*-KO mice (B6 background with the *znt7*-null gene from chromosome 3 of the 129P2 allele). Subcongenic strains are commonly used for dissection of complex trait genes. These mice possess a genomic segment from a donor strain, which is transferred through recurrent backcrossing to the background of a recipient strain [25]. In the current study, the donor strain was the 129P1 and the recipient strain was the congenic B6^*znt7*-KO^. We first created a subcongenic mouse strain (B6.129P1-7L^*znt7*-KO^, where B6 = the C57BL/6 recipient strain; 129P1 = the 129P1/ReJ donor stain; 7 = chromosome 7; L = long genomic segment) containing a 129P1 donor region from chromosome 7 (rs13479104 - rs13479276; about 34.7-Mb long) in the B6 background using speed congenics. We also created a second subcongenic mouse strain (B6.129P1-7SD^*znt7*-KO^, where SD = short genomic segment distal) in which a distal part of the donor segment in the B6.129P1-7L^*znt7*-KO^ strain was retained (about 17.7-Mb in size) while the proximal portion became the B6 allele. We conducted a phenotyping study with the 2 subcongenic mouse strains (males) and compared the results with that of B6^*znt7*-KO^ mice. The result suggest that the genomic segment responsible for weight and glucose tolerance may be located in a 17-Mb region, which is the proximal portion of the previous mapped QTL [16]. We also present four candidate genes in this mapped genomic region for the regulation of weight, lipid deposit, and glucose tolerance using quantitative RT-PCR and cSNP detection (single nucleotide polymorphisms in the protein coding region).

## Results

### Generation of subcongenic mice and determination of the genomic border regions on chromosome 7

**Fig. 1:**
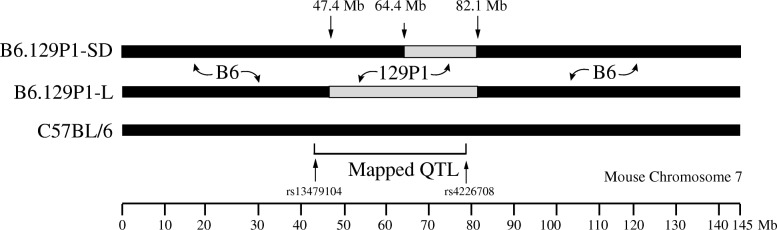
Subcongenic mapping. The 129P1 donor regions on mouse chromosome 7 in the subcongenic strain created by backcrossing male B6^*znt7*-KO^ congenic mice to female 129P1 mice are shown by the light grey bars. The black bars indicate the region from the B6 recipient genome. The genomic locations of the proximal and distal borders of the B6.129P1-7 L and B6.129P1-7SD regions were indicated above the bars. A previously identified QTL for body weight and fat mass [16] is presented under the bars. SNP markers used to define the QTL are listed. Physical map positions (Mb) of chromosome 7 are indicated on the *x*-axis according to the UCSC GRCm38/mm10 genome assembly. QTL, quantitative trait locus; BW, body weight; SNP, single nucleotide polymorphism; Mb, megabase

We previously reported a QTL on chromosome 7 in male *znt7*-KO mice that influenced weight gain and fat deposit [16]. This QTL encompasses a genomic region from 40.3 to 81.3 Mb on chromosome 7 with the region affecting fat deposit located close to the proximal end and the region affecting weight gain to the distal end. To confirm and fine-map the QTL, we generated two subcongenic mouse strains that carried different lengths of the genome segments on chromosome 7 from a 129P1 donor strain in the B6 recipient background. We created the subcongenic mouse strain by crossing male homozygous *znt7*-KO mice (congenic mice in the B6 background) to female 129P1 mice (Jackson Laboratories). After at least 8 generations of backcross to B6 with an assistance of a whole genome SNP genotyping panel [16], we first established a subcongenic mouse strain (B6.129P1-7L^*znt7*-het^) containing a 34.7-Mb 129P1-derived genomic segment of chromosome 7 (47.4–82.1 Mb based on the Mouse GRCm38/mm10 Assembly). The second subcongenic mouse strain (B6.129P1-7SD^*znt7*-het^) containing a 17.7-Mb genomic segment (64.4–82.1 Mb) from the 129P1 chromosome 7 was obtained by back-crossing male B6.129P1-7L^*znt7*-het^ to female B6 mice (Fig. 1). The proximal and distal breaking points of the 129P1 donor genomic segments in the two subcongenic mice were determined by genomic DNA sequencing and the genomic locations were determined according to the University of California Santa Cruz (UCSC) GRCm38/mm10 genome assembly (Fig. 1 and Table 1).

**Table 1:** Subcongenic strains

Strains	Genetic background	Donor strain	Chromosome	Size of the donor segment (Mb)	Physical location of the donor segment (Mb)
C57BL/6	C57BL/6J	n.a.	n.a.	n.a.	n.a.
B6.129P1-L	C57BL/6J	129P1/ReJ	7	34.7	47.4–82.1
B6.129P1-SD	C57BL/6J	129P1/ReJ	7	17.7	64.4–82.1

All strains carried the *znt7*-null alleles on Chr. 3 from a 129P2 strain; *Chr*. chromosome, *L* long donor genetic segment, *SD* short donor genetic segment distal, *Mb* megabase, *n.a*. not applicable

### Phenotyping subcongenic mice

**Fig. 2:**
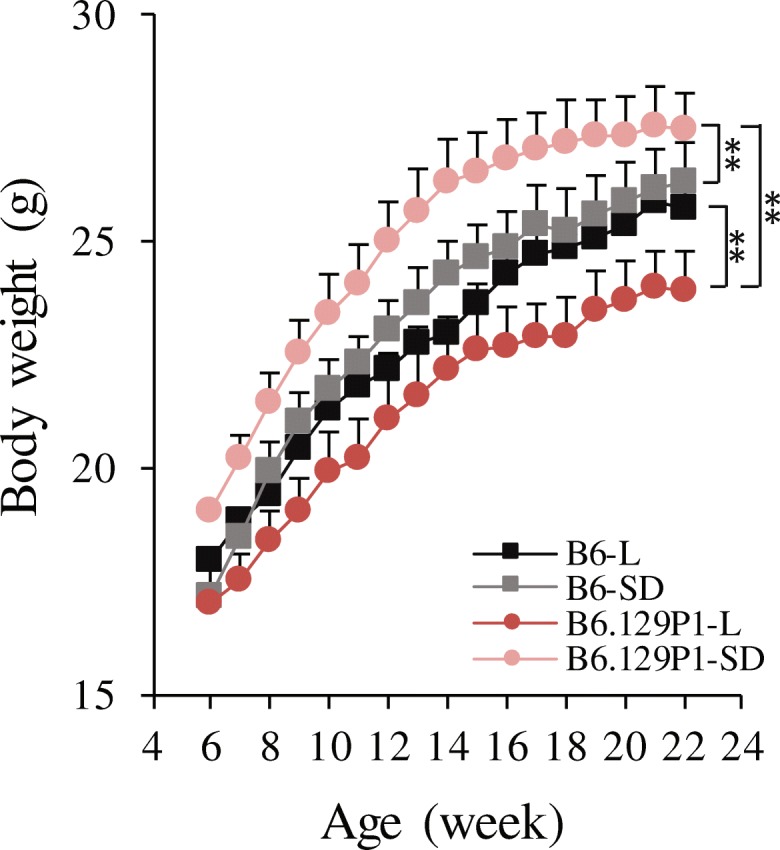
Growth curves of subcongenic and control mice. Mice were fed a semi-purified diet containing 30 mg zinc/kg diet from 5 to 24 weeks of age. Body weight was measured weekly. All values are expressed as mean ± S.E. (*error bars*), *n* = 7–9/group. **, *p* < 0.01. 129P1-L, subcongenic mice with a 34.7-Mb 129P1 donor region in the B6 background; 129P1-SD, subcongenic mice with a 17.7-Mb 129P1 donor region (the distal part of the 129P1-L allele) in the B6 background; B6-L and B6-SD, the C57BL/6 controls for 129P1-L and 129P1-SD subcongenic mice, respectively. All experimental animals were *znt7*-KO. Mb, megabase

Subcongenic mice heterozygous for *znt7*-KO and B6^*znt7*-het^ control mice were inbred by sister-brother matings. Male B6.129P1-7L^*znt7*-KO^, B6.129P1-7SD^*znt7*-KO^, and B6^*znt7*-KO^ mice were subsequently used for determining the effect of the 129P1 donor allele on weight gain and glucose metabolism. We used male mice for this study as our previous study demonstrated that male *znt7*-KO mice presented more noticeable phenotypes in growth and glucose metabolism than females [14]. The experimental animals were fed a semi-purified diet containing 30 mg zinc (from zinc carbonate)/kg diet at 5 weeks of age. We used this diet to control dietary zinc intake in mice as zinc contents and sources varied in regular mouse chow diets. Body weights were monitored weekly. As shown in Fig. 2, compared to the B6^*znt7*-KO^ control mice, B6.129P1-7L^*znt7*-KO^ mice had approximate 9% reduction in body weights (*p* < 0.03) at 22 weeks of age. On average, B6.129P1-7L^*znt7*-KO^ mice weighed 23.9 g (S.E. = 0.9, *n* = 7) while B6^*znt7*-KO^ mice weighed 26.3 g (S.E. = 0.6, *n* = 9) at 22 weeks of age. On the other hand, when compared the body weight of B6.129P1-7SD^*znt7*-KO^ mice to that of the B6^*znt7*-KO^ control mice, B6.129P1-7SD^*znt7*-KO^ mice appeared to grow slightly faster than the B6^*znt7*-KO^ control mice, especially during the early and middle growth phases, i.e., 6 to 14 weeks of age (*p* < 0.01) (Fig. 2). This effect was more obvious when comparing the growth curve of B6.129P1-7L^*znt7*-KO^ to that of B6.129P1-7SD^*znt7*-KO^ (Fig. 2). These results suggest that the 129P1 donor genomic segment in the distal region of the QTL, ranging from 64.4 to 82.1 Mb on chromosome 7, could positively influence the growth of *znt7*-KO mice while the 129P1 donor genomic segment in the proximal region (47.4–64.4 Mb) might negatively affect the weight gain of the *znt7*-KO mice.

### Glucose metabolism in the subcongenic mice

**Fig. 3:**
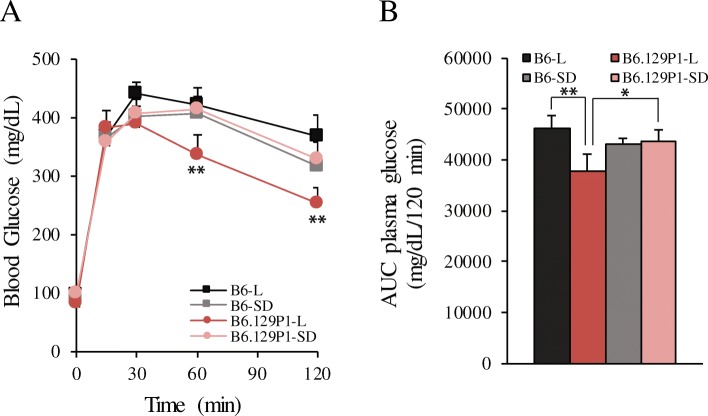
Blood glucose levels during intraperitoneal glucose tolerance test (IPGTT) and the area under the curve (AUC) of the blood glucose levels. **a** IPGTT. **b** The AUC of plasma glucose levels during IPGTT. Mice were fed a semi-purified diet containing 30 mg zinc/kg diet from 5 to 24 weeks of age. IPGTT was performed at 23 weeks of age. All values are expressed as mean ± S.E. (*error bars*), *n* = 7–9/group. *, *p* < 0.05; **, *p* < 0.01. 129P1-L, subcongenic mice with a 34.7-Mb 129P1 donor region in the B6 background; 129P1-SD, subcongenic mice with a 17.7-Mb 129P1 donor region (the distal part of the 129P1-L allele) in the B6 background; B6-L and B6-SD, the C57BL/6 controls for 129P1-L and 129P1-SD subcongenic mice, respectively. All experimental animals were *znt7*-KO. Mb, megabase

**Fig. 4:**
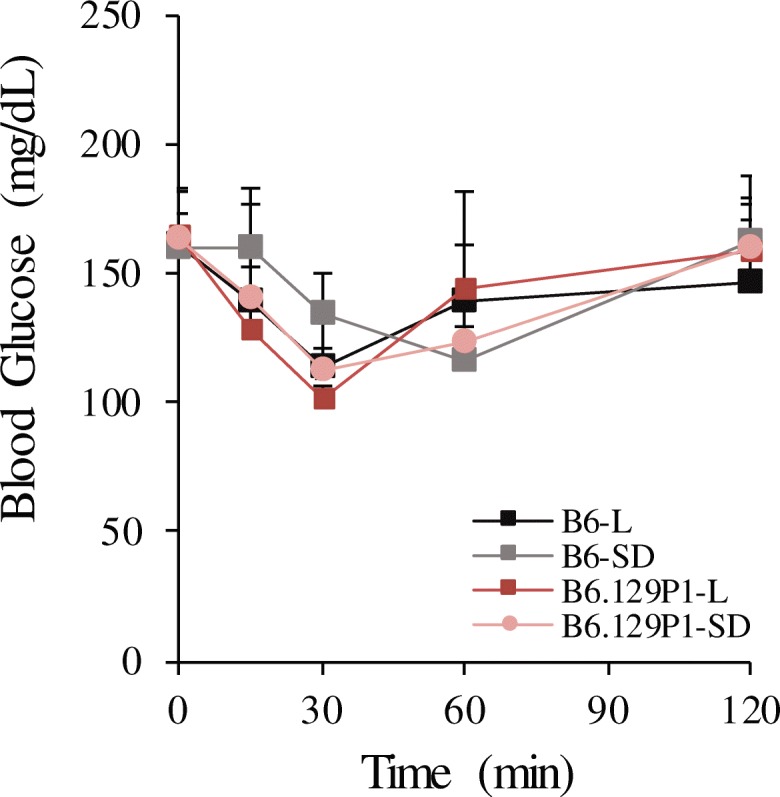
Blood glucose levels during intraperitoneal insulin tolerance test (IPITT). Mice were fed a semi-purified diet containing 30 mg zinc/kg diet from 5 to 24 weeks of age. IPITT was performed at 22 weeks of age. All values are expressed as mean ± S.E. (*error bars*), *n* = 7–9/group. 129P1-L, subcongenic mice with a 34.7-Mb 129P1 donor region in the B6 background; 129P1-SD, subcongenic mice with a 17.7-Mb 129P1 donor region (the distal part of the 129P1-L allele) in the B6 background; B6-L and B6-SD, the C57BL/6 controls for 129P1-L and 129P1-SD subcongenic mice, respectively. All experimental animals were *znt7*-KO. Mb, megabase

We previously demonstrated that male *znt7*-KO mice had impaired glucose tolerance and were prone to diet-induced insulin resistance in the B6 genetic background [13, 14]. Thus, in the current study, we investigated whether the 129P1-derived genomic segment from chromosome 7 had an effect on glucose metabolism in the B6.129P1-7L^*znt7*-KO^ and B6.129P1-7SD^*znt7*-KO^ subcongenic mice compared to that of the B6^*znt7*-KO^ control mice. Intraperitoneal insulin tolerance test (IPITT) and intraperitoneal glucose tolerance test (IPGTT) were performed in these mice at 22 and 23 weeks of age, respectively. As shown in Fig. 3a & b, *znt7*-KO mice carrying the full length of the 129P1-derived QTL (B6.129P1-7L^*znt7*-KO^) were more tolerant to glucose challenge during IPGTT than the B6^*znt7*-KO^ control mice. Whereas, *znt7*-KO mice carrying the distal portion of the QTL (B6.129P1-7SD^*znt7*-KO^) had similar blood glucose levels to that of the B6^*znt7*-KO^ control mice during IPGTT. When compared the blood glucose levels of B6.129P1-7L^*znt7*-KO^ mice during IPGTT to that of B6.129P1-7SD^*znt7*-KO^ mice, B6.129P1-7L^*znt7*-KO^ mice had a better blood glucose control. Nevertheless, we did not detect any differences in the peripheral glucose usage during IPITT among B6.129P1-7L^*znt7*-KO^, B6.129P1-7SD^*znt7*-KO^, and B6^*znt7*-KO^ mice (Fig. 4), suggesting that peripheral glucose deposition was not affected by the 129P1 donor allele. Taken together, these results suggest the existence of at least a candidate gene for glucose control in the proximal region of the QTL from the 129P1 donor allele.

### Gene expression in the proximal 129P1 donor region

**Fig. 5:**
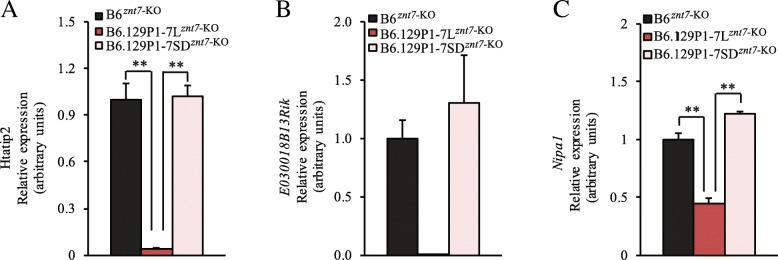
mRNA expression of *Htatip2*, *E030018B13Rik*, and *Nipa1* in the subcutaneous fat samples. **a** *Htatip2* mRNA expression. **b** *E030018B13Rik* mRNA expression. **c** *Nipa1* mRNA expression. Total RNAs were purified from the subcutaneous fat of B6.129P1-7L^*znt7*-KO^, B6.129P1-7SD^*znt7*-KO^, and the B6^*znt7*-KO^ control mice at necropsy. The quantity of the target mRNA was measured by a SYBR-based quantitative RT-PCR. *Actb* was used as an internal reference. Results are presented as mean ± S.D. (*n* = 4/genotype). **, *p* < 0.01

We showed that the proximal 129P1 donor region on chromosome 7 displayed beneficial effects on weight gain and glucose control during IPGTT (Fig. 3). We, therefore, had focused on the candidate gene search in this region. The proximal donor region ranged from 47.4 to 64.4 Mb on chromosome 7 based on our genome sequencing results of B6.129P1-7L^*znt7*-KO^ and B6.129P1-7SD^*znt7*-KO^ mice (Fig. 1 and Table 1). A total of 53 protein coding genes are located in this donor region according to the mouse genome GRCm38p assembly (Table 2). Among them, 13 are Mrgp members (Mas-related G-protein coupled receptor), including *Mrgpra1*, *Mrgpra3*, *Mrgpra4*, *Mrgpra2a*, *Mrgpra2b*, *Mrgprb1*, *Mrgprb2*, *Mrgprb3*, *Mrgprb4*, *Mrgprb5*, *Mrgprb8*, *Mrgprx1*, and *Mrgprx2*, and 3 Gaba A receptor subunits (gamma-aminobutyric acid), including *Gabra5*, *Gabrb3* and *Gabrg3*. These genes have been reported to be exclusively or predominantly expressed in the mouse nervous system [26, 27]. Therefore, we excluded these 16 genes in our quantitative gene expression analysis using subcutaneous adipose tissue samples isolated from subcongenic and control mice. For the remaining 37 genes, we first obtained mRNA expression information in the mouse adipose tissue of the B6 strain by searching the Mouse ENCODE Transcriptome database (https://www.ncbi.nlm.nih.gov/bioproject/PRJNA66167/). As shown in Table 2, based on the database, the majority of genes (24 of them) were expressed in the subcutaneous fat of B6 mice. The expression of 7 other genes (*Csrp3*, *Dbox1*, *Slc6a5*, *Slc17a6*, *1700015G11Rik*, *Luzp2*, and *Oca2*) was not detected in the subcutaneous fat of B6 mice. We also excluded these 7 genes in our quantitative RT-PCR assays. For the remaining of 6 genes, there was no gene expression information found in the Mouse ENCODE Transcriptome database, including *4933405O20Rik*, *Fancf*, *Ndn*, *Magel2*, *Mkrn3*, and *Pegl2*. Therefore, we included these 6 genes in our quantitative RT-PCR assays. Thus, we investigated mRNA expression of a total of 30 genes in the subcutaneous fat samples isolated from B6.129P1-7L^*znt7*-KO^ and B6^*znt7*-KO^ mice and compared the gene expression levels between the two strains. We chose to examine mRNA expression in the subcutaneous fat as we previously reported that lipid and glucose metabolism in this tissue was negatively affected by the *znt7*-null mutation while this negative effect was not observed in the epididymal fat of *znt7*-KO mice [17]. The results indicated that only three genes, including *Htatip2, E030018B13Rik,* and *Nipa1* displayed a differential expression pattern between B6.129P1-7L^*znt7*-KO^ and B6^*znt7*-KO^ mice. We further analyzed the mRNA expression of these three genes to include the subcutaneous fat tissue from B6.129P1-7SD^*znt7*-KO^ mice. As shown in Fig. 5, expression of *Htatip2* and *Nipa1* was down-regulated by 95.6% (*p* < 0.01) and 49% (*p* < 0.05) in the subcutaneous fat from B6.129P1-7L^*znt7*-KO^ mice, respectively, compared to that from the B6^*znt7*-KO^ control mice. No difference in mRNA expression of *Htatip2* and *Nipa1* between B6.129P1-7SD^*znt7*-KO^ and B6^*znt7*-KO^ control mice was observed. Furthermore, little to no mRNA was detected for *E030018B13Rik* in the subcutaneous fat from B6.129P1-7L^*znt7*-KO^ mice. However, the *E030018B13Rik* mRNA was readily detected in the subcutaneous fat from both B6.129P1-7SD^*znt7*-KO^ and B6^*znt7*-KO^ control mice (Fig. 5). In addition, no statistically significant difference in the mRNA expression of *E030018B13Rik* was noticed between B6.129P1-7SD^*znt7*-KO^ and B6^*znt7*-KO^ control mice. Taken together, the results suggest that the differential mRNA expression of *Htatip2, E030018B13Rik,* and *Nipa1* in the subcutaneous fat was only limited to B6.129P1-7L^*znt7*-KO^ but not B6.129P1-7SD^*znt7*-KO^ mice.

**Table 2:** Gene expression in *Znt7*-KO/129P1-L proximal congenic region (47.4–64.4 Mb)

Mb	Gene Symbol	Gene Name	mRNA expression in SC fat (RPKM)^†^	mRNA expression in SC fat (RT-PCR)	Differential expression
48.78	Zdhhc13	zinc finger, DHHC domain containing 13	3.564	yes	no
48.83	Csrp3	cysteine and glycine-rich protein 3	N.D.	–	–
48.86	E2f8	E2F transcription factor 8	0.631	yes	no
48.95	Nav2	neuron navigator 2	0.803	yes	no
49.63	Dbx1	developing brain homeobox 1	N.D.	–	–
49.75	Htatip2	HIV-1 tat interactive protein 2	5.552	yes	yes**
49.77	Prmt3	protein arginine N-methyltransferase 3	5.464	yes	no
49.90	Slc6a5	solute carrier family 6 (neurotransmitter transporter, glycine), member 5	N.D.	–	–
49.97	Nell1	NEL-like 1	0.941	yes	no
50.59	4933405O20Rik	RIKEN cDNA 4933405O20 gene	n/a	yes	no
51.51	Ano5	anoctamin 5	0.126	yes	no
51.62	Slc17a6	solute carrier family 17 (sodium-dependent inorganic phosphate cotransporter), member 6	N.D.	no	–
51.86	Fancf	Fanconi anemia, complementation group F	n/a	no	–
51.86	Gas2	growth arrest specific 2	0.608	yes	no
51.99	Svip	small VCP/p97-interacting protein	1.731	yes	no
52.01	1700015G11Rik	RIKEN cDNA 1700015G11 gene	N.D.	–	–
54.83	Luzp2	leucine zipper protein 2	N.D.	–	–
55.76	Siglech	sialic acid binding Ig-like lectin H	0.609	yes	no
55.79	Tubgcp5	tubulin, gamma complex associated protein 5	1.234	yes	no
55.84	Cyfip1	cytoplasmic FMR1 interacting protein 1	9.187	yes	no
55.93	Nipa2	non imprinted in Prader-Willi/Angelman syndrome 2 homolog (human)	2.822	yes	no
55.97	Nipa1	non imprinted in Prader-Willi/Angelman syndrome 1 homolog (human)	4.38	yes	yes*
56.05	Herc2	hect (homologous to the E6-AP (UBE3A) carboxyl terminus) domain and RCC1 (CHC1)-like domain (RLD) 2	5.275	yes	no
56.23	Oca2	oculocutaneous albinism II	N.D.	no	–
58.65	Atp10a	ATPase, class V, type 10A	4.05	yes	no
59.22	Ube3a	ubiquitin protein ligase E3A	2.997	yes	no
59.98	Snurf	SNRPN upstream reading frame	9.687	yes	no
59.98	Snrpn	small nuclear ribonucleoprotein N	8.022	yes	no
62.34	Ndn	necdin	n/a	yes	no
62.37	Magel2	melanoma antigen, family L, 2	n/a	yes	no
62.41	Mkrn3	makorin, ring finger protein, 3	n/a	yes	no
62.46	Peg12	paternally expressed 12	n/a	yes	no
63.09	Chrna7	cholinergic receptor, nicotinic, alpha polypeptide 7	0.121	yes	no
63.44	Otud7a	OTU domain containing 7A	0.017	yes	no
63.88	Klf13	Kruppel-like factor 13	16.442	yes	no
63.91	E030018B13Rik	RIKEN cDNA E030018B13 gene	0.147	yes	yes**
64.15	Trpm1	transient receptor potential cation channel, subfamily M, member 1	0.036	no	–

^†^Mouse ENCODE transcriptome data (https://www.ncbi.nlm.nih.gov/bioproject/PRJNA66167/); A cluster of brain-specific expressed Mrgp (MAS-related GPR) genes (47.33–48.64 Mb) are not listed in the Table, including Mrgpra1, Mrgpra3, Mrgpra4, Mrgpra2a, Mrgpra2b, Mrgprb1, Mrgprb2, Mrgprb3, Mrgprb4, Mrgprb5, Mrgprb8, Mrgprx1, and Mrgprx2; *RPKM* reads per kilobase per million reads placed, *SC* Subcutaneous fat, *N.D.* not detected, *n.d.* not determined, *n/a* not available; *p < 0.05; **p < 0.01 (B6.129P1-7L^*Znt7*-KO^ vs B6 ^*Znt7*-KO^)

### Sequence analysis of coding regions in the proximal 129P1 donor region

Since variations in the protein coding sequence that changes an amino acid can be deleterious for protein structures and/or functions independent of mRNA expression, we analyzed the coding sequences for all 37 genes, except for the members of Mrgp and Gaba A receptors, in the proximal 129P1 donor region of the QTL. We found a nonsense mutation in both *Oca2* and *E030018B13Rik* of the 129P1 allele changing an amino acid to a stop codon that could result in nonsense-mediated mRNA decay and the coupled premature termination of translation (Table 3). We also found a non-synonymous change in the Atp10a protein sequence (a glycine residue in the B6 allele is substituted by the cysteine residue in the 129P1 allele at the amino acid position 40). This amino acid change was predicted to be deleterious by the SIFT (Sorting Intolerant from Tolerant) algorithms (http://sift.bii.a-star.edu.sg/) (Table 3). This deleterious amino acid change was also supported by the Polyphen-2 program with a score close to 1 (while scores close to 0 are predicted to be benign and scores to 1 indicate intolerance to substitution (http://genetics.bwh.harvard.edu/pph2/).

**Table 3:** Non-tolerant amino acid changes in the genes located in the B6.129P1-7L^*Znt7*-KO^ proximal region (47.4–64.4 Mb)^a^

Gene Symbol	cSNP	SNP ID	Protein Effect	PolyPhen-2 score^b^	Transcript ID
Oca2	c.784C > T	rs32980941	p.Arg262Ter	n.a.	ENSMUSP00000032633.5
Atp10a	c.118G > T	rs240949563	p.Gly40Cys	0.998	ENSMUST00000168747.2
E030018B13Rik	c.247G > T	rs31348450	p.Gly83Ter	n.a.	ENSMUST00000177638.1

^a^non-tolerant amino acid change was predicted by SIFT (Sorting Intolerant from Tolerant, http://sift.bii.a-star.edu.sg/www/SIFT_seq_submit2.html); ^b^http://genetics.bwh.harvard.edu/pph2/index.shtml. Scores close to 0 are predicted to be benign and scores to 1 indicate intolerance to substitution; *n.a.* not applicable

## Discussion

Our subcongenic mouse analysis with *znt7* deficiency was prompted by the observation that the *znt7*-KO in a mixed B6 and 129P2 genetic background displayed a greater reduction in body weight and fat deposit than the *znt7*-KO on the B6 background [13]. A subsequent genome-wide mapping study revealed a single significant QTL for weight gain and fat deposit using F2 male mice with a B6.129P1^*znt7*-KO^ and B6^*znt7*-KO^ mixed background [16]. This QTL ranges from 40.3 to 81.3 Mb on mouse chromosome 7. In this study, we employed a subcongenic breeding strategy aimed at conformation of our previous discovery of *znt7*-KO interacting with a gene on mouse chromosome 7 and, importantly, reduction in the size of the QTL to positionally locate a candidate gene. We confirmed the linkage of the QTL to weight regulation in subcongenic mice. We also successfully narrowed this QTL down to a candidate genomic region of 17-Mb located in the proximal portion of the QTL on chromosome 7. We demonstrated that the sub-QTL (47.4–64.4 Mb) from the 129P1 allele conferred lower weight gain in B6.129P1-7L^*znt7*-KO^ mice than the B6^*znt7*-KO^ controls (Fig. 2). Moreover, we showed that this sub-QTL from the 129P1 donor allele contributed to a better glucose control during glucose tolerance test. Based on our previous results obtained from body composition and fat pad deposit analyses in *znt7*-KO mice [13, 16], the reduced body weights observed in male B6.129P1-7L^*znt7*-KO^ mice is likely to reflect the change in fat deposit. Taken together, our findings strongly suggest the presence of an obese protective gene in the 129P1 donor allele on mouse chromosome 7.

Remarkably, in this study, we observed that mice carrying the 129P1 donor allele from the distal portion of the QTL (64.4–82.1 Mb) conferred a positive effect on weight phenotype without affecting diabetes-related phenotypes, such as IPGTT profile, area under the curve during IPGTT, and insulin response in *znt7*-KO mice. It was surprising because the full QTL (47.4–82.1 Mb) from the 129P1 allele displayed a negative effect on body weight and fat mass (Fig. 2) [16]. According to the QTL mapping result [16], the QTL peak for the reduced weight gain from the 129P1 allele is at 73.5 Mb and the peak for the reduced fat deposit is at 70.3 Mb. Unexpectedly, the proximal portion of the QTL, which conferred lower body weight and improved glucose control during IPGTT, did not include the two QTL peaks. This phenomenon could be explained by the presence of at least two QTLs affecting body weight and/or adiposity in the originally mapped QTL. The impacts of the two QTLs on weight gain and/or fat deposit counteract each other. When both of the alleles appear together in a larger genomic region, their opposite effect on growth and/or fat mass negate each other or one allele may present stronger effect than the other. In this case, it appears that the sub-QTL of the 129P1 allele in the proximal region of the QTL presented stronger effect on weight than the one in the distal region of the QTL. Therefore, the effect from the QTL of the 129P1 allele (B6.129P1-7L^*znt7*-KO^) showed a negative effect on growth. Whereas when the counteracting QTLs were separated, such as B6.129P1-7SD^*znt7*-KO^ mice, the underlying individual sub-QTL’s effect became obvious. As a result, the two counteractive alleles presented in one segment of chromosome 7 could lead to shifts of the QTL peaks. Indeed, the presence of opposite effect of QTLs on obesity was reported previously [28]. Diament and Warden have demonstrated that at least three QTLs regulating obese phenotype are located on mouse chromosome 7 [28]. It is apparent that there are multiple loci on chromosome 7 that modulate body weight and fat mass in mice. We believe that a protective locus against obesity and diabetes is present in the 129P1 allele at the proximal region of the QTL on chromosome 7 although creating a subcongenic mouse line carrying the proximal portion of the QTL region may be necessary for further confirmation. In addition, an obesity susceptibility gene in the distal region retained in B6.129P1-7SD^*znt7*-KO^ mice cannot be excluded.

In the mapped proximal region of the QTL, 53 protein-coding genes are located with 2 clusters of brain-enriched genes, Mas-related G-protein coupled receptors (13 genes) and Gaba A receptor subunits (3 genes). Of the remaining 37 protein-coding genes, mRNA expression could be readily detected for 28 genes in the subcutaneous fat isolated from B6.129P1-7L^*znt7*-KO^ and B6^*znt7*-KO^ mice (Table 2) using quantitative RT-PCR. Among these 28 genes, 3 genes, including *Htatip2*, *E030018B13Rik*, and *Nipa1*, displayed differential mRNA expression between the 129P1 donor allele and the B6 recipient allele. Little to no mRNA expression for the remaining 9 genes, including *Csrp3*, *Dbx1*, *Slc6a5*, *Slc17a6*, *Fancf*, *1700015G11Rik*, *Luzp2*, *Oca2*, and *Trpm1*, was detected in the fat from B6.129P1-7L^*znt7*-KO^ and B6^*znt7*-KO^ mice using our current detection method or results from the Mouse ENCOD Transcriptome database. Thus, based on our quantitative mRNA expressing results, we propose that *Htatip2*, *E030018B13Rik*, and *Nipa1* may likely be the candidates as modifier genes for *znt7*-KO modulating body weight and fat mass in the mouse genome.

The mRNA expression of *Htatip2*, a gene encoding an oxidoreductase, was greatly suppressed (> 95%) from the 129P1 allele compared to the B6 allele. *Htatip2* is ubiquitously expressed in mouse and human tissues and plays an inhibitory role in regulation of nuclear import [29]. It may function as a negative regulator by competing with nuclear import substrates for nuclear transport receptor binding [30]. This inhibitory role may facilitate Htatip2 as a tumor repressor since down-regulation of *Htatip2* is associated with development of a number of cancer and/or induction of aggressive metastasis, including lung cancer [31], hepatocarcinoma [32, 33], breast cancer [34], brain tumor [35]. Interestingly, recent studies have identified roles of Htatip2 in regulation of metabolism. It has been shown that silencing *Htatip2* expression in tumor cells, such as MCF7 and HeLa cells, can largely increase metabolic flexibility, i.e. increased mitochondria activity to produce energy to meet the requirement for cell survival and proliferation during glucose restriction due to high levels of both glycolysis and mitochondrial respiration in tumor cells [36]. One of the explanations for this observation is that silencing *Htatip2* expression may stimulate usage of substrates, such as fatty acids and amino acids [37]. Htatip2 is detected in a protein complex required for cellular vesicle membrane fusion [32]. Acsl4 (acyl-Co A synthetase long-chain family member 4), an enzyme converting free long-chain fatty acids into fatty acyl-CoA esters, is also a component of this protein complex. Acsl4 plays a critical role in fatty acid oxidation and lipid biosynthesis [38]. Moreover, Liao et al. revealed that the expression of Htatip2 was significantly down-regulated in the liver of *Pkcδ*-KO mice fed a high fat diet [37]. The authors further addressed that Htatip2 was involved in lipid droplet formation in HepG2 hepatocytes and knockdown of *Htatip2* decreased the incorporation of fatty acids into triglycerides in IHH (immortalized human hepatocytes) [37]. It is suggested that Htatip2 may be involved in determining the partitioning between lipid storage and oxidation. Moreover, *Htatip2* is a target gene regulated by Nfe2l2 (nuclear factor, erythroid derived 2, like 2), a transcription factor which regulates genes containing antioxidant response elements in their promoters [39]. Mice deficient for *Nfe2l2* have impaired adipogenesis and are protected from diet-induced obesity [40]. Recently, we have demonstrated that ZnT7 regulates lipid partitioning in mice by increasing lipid storage in adipocytes while *znt7*-KO reduces lipid formation in adipocytes. *znt7*-KO also stimulates fatty acid uptake and oxidation in skeletal muscle [15, 17]. *znt7*-KO accompanied with reduced *Htatip2* expression from the 129Pl allele may exert a synergistic effect on lipid metabolism in adipose tissue, leading to a lean phenotype in mice. It is worth noting that down-regulation of *Htatip2* in B6.129P1-7L^*znt7*-KO^ mice also improved glucose handling during glucose tolerant test. However, the mechanism underlying this phenotype is currently not understood and warrants further studies.

Four other candidates in the sub-QTL region were also discovered by quantitative RT-PCR, DNA sequencing, and cSNP analysis. First, we found that the *E030018B13Rik* transcript was down-regulated by 83% in the fat tissue from B6.129P1-7L^*znt7*-KO^ mice compared to the B6^*znt7*-KO^ control. Further DNA sequencing and cSNP analysis revealed the presence of a nonsense mutation in the *E030018B13Rik* gene of the 129P1 allele converting glycine at the amino acid position 84 to a pre-mature stop codon. The *E030018B13Rik* gene encodes an unknown function protein with 113 amino acids and this gene is widely expressed in mouse tissues according to the Mouse ENCODE transcriptome database. Second, we detected that *Nipa1* (non-imprinted in Prader-Willi/Angelman syndrome 1 homolog (human)), a gene that encodes a 323 amino acid long protein, was down-regulated by ~ 50% from the 129P1 allele in B6.129P1-7L^*znt7*-KO^ mice compared to the control. *Nipa1* mRNA is ubiquitously expressed in mouse tissues (the Mouse ENCODE transcriptome database). Mutations in the human *NIPA1* gene cause an autosomal dominant hereditary spastic paraplegia (HSP), a neurodegenerative disorder characterized by progressive lower limb spasticity and weakness [41]. Goytain et al. demonstrated that NIPA1 was localized in early endosomes as well as on the cell surface in a variety of neuronal and epithelial cells, which facilitates Mg_2_^++^ transport across the membrane. Although the mRNA expression of *Nipa1* was down-regulated about 50% in the fat tissue of B6.129P1-7L^*znt7*-KO^ mice, we did not observe any neuronal abnormalities or limb weakness in these animals during our study. Third, the *Oca2* gene encodes a melanocyte-specific transporter protein, also called P protein. Mutations in the human *OCA2* result in oculocutaneous albinism II and, in the mouse gene, a mutation leading to a premature stop codon in the protein that causes a pink-eyed dilution mutant. The P protein is thought to be involved in small molecule transport, including tyrosine. B6.129P1-7L^*znt7*-KO^ mice carried the *p* mutation (amino acid 262, p.Arg262Ter) which was evident by our genomic DNA sequencing. Nevertheless, we could not detect either *Oca2* mRNA or protein in the subcutaneous fat tissues isolated from both B6.129P1-7L^*znt7*-KO^ and B6^*znt7*-KO^ mice (Table 2 and data not shown). Lastly, our genomic DNA sequencing analysis demonstrated the presence of a non-synonymous change in the Atp10a protein sequence at the amino acid position 40 (p.Gly40Cys, B6/129P1). This amino acid change is predicted to be deleterious to the protein function. *Atp10a* mRNA is ubiquitously expressed in mouse tissues and the protein is localized on the cell surface [42, 43]. It functions as an ATP-dependent aminophospholipid translocase regulating lipid compositions at the plasma membrane, such as phosphatidylcholine (PC). Change in Atp10a activity can affect cell shape and mobility [44]. Atp10a was reported to be the candidate gene for body fat regulation on mouse chromosome 7 [43]. Heterozygous deletions of *Atp10a* in mice lead to diet-induced obesity, insulin resistance, and nonalcoholic fatty liver disease [45]. Atp10a is also implicated in regulation of insulin-stimulated glucose uptake via regulation of the MAPK signaling pathway in skeletal muscle and adipose tissue [46, 47]. Nevertheless, our insulin tolerance tests revealed that the peripheral insulin sensitivity seemed not to be affected by the QTL containing the Atp10a gene (Fig. 4).

## Conclusions

We had successfully narrowed down the QTL of body weight to a sub-QTL region of 17 Mb on chromosome 7 using two newly-created subcongenic mouse strains. We identified four candidates as the phenotype modifier genes for *znt7*-KO, including *Htatip2*, *E030018B13Rik*, *Nipa1*, and *Atp10a*, using quantitative RT-PCR, genomic DNA sequencing, and cSNP analysis. Significantly differential expression of *Htatip2* in the subcutaneous fat and its function in regulation of lipid metabolism makes it the best candidate among the four candidate genes. Further studies are needed to confirm the genetic effect of the proximal region of the QTL on weight and glucose tolerance in subcongenic mice carrying this genomic segment and characterize candidate genes, especially *Htatip2*, in regulation of weight gain, fatness, and glucose control associated with zinc metabolism.

## Methods

### Mouse strains, husbandry, and diets

The congenic *znt7*-KO mouse strain on the B6 genetic background was described previously [13]. 129P1/ReJ mice were purchased from the Jackson Laboratory (Bar Harbor, ME). Male *znt7*-KO mice were mated to female 129P1 mice and the resulting heterozygous male *znt7*-KO mice were backcrossed to B6 female mice (Jackson Laboratory). After 3 generations of backcrossing, male *znt7*^+/−^ mice were genotyped with a genome-wide SNP panel [16] to select those with a high-coverage of the B6 genome for the next generation backcross to B6 except for Chromosome 7, which we selected for the 129P1 allele between SNP markers, rs45883751 (43.6 Mb) and rs4226708 (79.0 Mb). This procedure was repeated for 3 generations until all markers in the panel displayed the B6 allele except for chromosome 7. We performed two final backcrosses after we selected the subcongenic *Znt7*^+/−^ mice that carried a 129P1 genomic segment containing the following SNP markers on chromosome 7, rs45883751 (43.6 Mb), rs32166708 (64.2 Mb), rs13479335 (73.5 Mb), and rs4226708 (79.0 Mb). We named this subcongenic mouse strain as B6.129P1-7L^*znt7-*het^ where B6 represents the background strain, 129P1 is the donor, 7 is the chromosome selected from the donor strain, L represents a large segment of the chromosome selected, and *znt7*^*-*het^ means that the strain carries a heterozygous *znt7*-null mutation on chromosome 3. B6^*znt7-*het^ background control mice were also selected during the last backcross. For this study, B6.129P1-7L^*znt7-*het^ or B6^*znt7-*het^ mice were intercrossed to obtain B6.129P1-7L^*znt7*-KO^ or B6^*znt7*-KO^ mice.

The second subcongenic strain, B6.129P1-7SD^*znt7-*het^ that carries a distal portion of the donor allele in B6.129P1-7L^*znt7-*het^, was generated by backcrossing male B6.129P1-7L^*znt7-*het^ mice to female B6 and selected by three SNP markers, rs32166708 (64.2 Mb), rs13479355 (73.5 Mb), and rs4226708 (79.0 Mb). SD indicates a small and distal portion of the donor allele being selected. We ended the backcross procedure when the rs32166708 SNP marker became the B6 allele whereas the other two SNP markers remained as the 129P1 allele. Finally, B6.129P1-7SD^*znt7-*het^ mice were intercrossed to obtain B6.129P1-7SD^*znt7-*KO^ and the B6 control mice.

Mice with desired genotypes were weaned at 3 weeks of age and fed a semi-purified diet containing 30 mg Zn/kg diet (Research Diets, New Brunswick, NJ) ad libitum from 5 to 24 weeks of age [13]. All mice were housed in a temperature-controlled room at 22-24 °C with a 12 h light:dark cycle. Breeding mice were fed a standard laboratory chow diet (Laboratory Rodent Diet 5001, LabDiet, Brentwood, MO) and double-distilled water ad libitum. Mice were euthanized by cardiac puncture under general anesthesia (intraperitoneal injection of 100 mg/Kg ketamine (MWI Veterinary Supply, Boise, ID) and 10 mg/Kg xylazine (MWI Veterinary Supply)). Euthanasia was confirmed by cervical dislocation and tissues were then isolated and stored at -80 °C until use. All animal experiments were conducted in accordance with National Institutes of Health guidelines for the Care and Use of Experimental Animals and were approved by the Institutional Animal Care and Use Committee of the University of California at Davis.

### Tail clipping, genomic DNA isolation and *znt7* genotyping

Tail tips were clipped from mice at around two-weeks-old. Genomic DNA was isolated from tail clips using a DNeasy Tissue kit (Qiagen, Valencia, CA). PCR genotyping to identify *znt7* heterozygotes or homozygotes was performed as described previously [16].

### Genome-wide SNP genotyping

A hundred polymorphic SNP markers informative between B6 and 129P1 strains were used for genotyping (TaqMan® SNP Genotyping Assays, TheromoFisher Scientific, Carlsbad, CA). All SNP markers used in this study and their genomic positions (GRCm38) were reported previously [16]. TaqMan® genotyping reactions were performed on QuantStudio 7 Flex according to the manufacturer’s instructions and allelic discriminations were performed using QuantStudio™ Real-time PCR software (TheromoFisher Scientific). All ambiguous genotypes were repeated in independent PCR reactions.

### Phenotyping

Only male mice were used in this study as the phenotype of body weight was more apparent in the *znt7*-KO males than the *znt7*-KO females [13, 14]. Body weights were measured from 6 to 22 weeks of age in the morning (~ 07:00, light phase of the day). Intraperitoneal insulin tolerance tests and intraperitoneal glucose tolerance tests were performed at 22 and 23 weeks of age, respectively. Mice were fasted overnight (16–18 h) and killed at 24 weeks of age [14]. Subcutaneous fat tissues were isolated, snap-frozen, and stored at -80 °C until use.

### Intraperitoneal glucose tolerance test (IPGTT)

Before the test, mice were fasted overnight. Glucose (1.5 g/kg of body weight) was given intraperitoneally. Blood glucose concentrations were determined at 0, 15, 30, 60, and 120 min after the glucose administration with a drop of blood from the tail vein using a One-Touch UltraMini meter (LifeScan, Chesterbrook, PA).

### Intraperitoneal insulin tolerance test (IPITT)

Before the test, mice were fasted for 4 h. Insulin (5.5 U of Humulin® R (U-100)/kg of body weight, Lilly, Indianapolis, IN) was given intraperitoneally. Blood glucose concentrations were determined at 0, 15, 30, 60, and 120 min after the insulin injection with a drop of blood from the tail vein using a One-Touch UltraMini meter (LifeScan).

### Genomic sequencing and analysis

Genomic DNA was purified from muscle and subjected to DNA sequencing. The library preparation and DNA sequencing (Illumina HiSeq4000) were carried by the DNA Technologies and Expression Analysis Cores at the UC Davis Genome Center supported by NIH Shared Instrumentation Grant 1S10OD010786–01 (http://dnatech.genomecenter.ucdavis.edu). The Bioinformatics Core Facility at the UC Davis Genome Center provided sequence data analysis (http://bioinformatics.ucdavis.edu). Coding SNPs (cSNPs) and their locations on chromosome 7 within the 129P1 donor region that differed from the B6 control were obtained using UCSC (University of California Santa Cruz) Genome Browser on the Mouse GRCm38/mm10 Assembly (https://genome.ucsc.edu/cgi-bin/hgGateway). The effect (tolerant or deleterious) of an amino acid substitution on protein function was predicted using both SIFT (Sorting Intolerant from Tolerant), which predicts possible effects based on sequence homology and the physical properties of amino acids (http://sift.bii.a-star.edu.sg/www/SIFT_seq_submit2.html), and PolyPhen-2 (Polymorphism Phenotyping v2), which predicts possible effects of an amino substitution on the structure and function of a protein using physical and comparative strategies (http://genetics.bwh.harvard.edu/pph2/index.shtml). Information about mRNA expression of the genes located in the proximal region of the 129P1 allele on chromosome 7 in the mouse subcutaneous fat tissue was obtained from the Mouse ENCORE transcriptome data (https://www.ncbi.nlm.nih.gov/bioproject/PRJNA66167/) (Table 1). We validated the majority of genes listed in Table 2 by quantitative RT-PCR using the subcutaneous fat samples purified from B6.129P1-7L^*znt7-*KO^, B6.129P1-7SD^*znt7-*KO^ and B6^*znt7*-KO^ mice in this study.

### Total RNA isolation, cDNA synthesis, and quantitative RT-PCR analysis

Approximately 50 mg frozen subcutaneous fat sample (*n* = 5–6/group) was lysed in 1 ml TRIzol (ThermoFisher Scientific). Total RNA was isolated according to the manufacturer’s instructions. One hundred ng total RNAs was converted into cDNAs using an iScript cDNA synthesis kit (BioRad). For quantitative RT-PCR analysis, synthesized cDNA was diluted 10-fold with double-distilled water, and 2 μl was used in a SYBR green-based PCR using SsoAdvanced™ SYBR® Green supermix (BioRad). Quantitative PCR was performed on a QuantStudio 7 Flex System (ThermoFisher Scientific). All primers used in this study are listed in Additional file 1: Table S1. The primer sequences were either obtained from the PrimerBank (https://pga.mgh.harvard.edu/primerbank/index.html) or designed using the Primer Design Tool (https://www.ncbi.nlm.nih.gov/tools/primer-blast). Primer oligos were synthesized by ThermoFisher Scientific. Melting temperature analysis for the reference (*Actb*) [14] and the target genes were analyzed. Quantitative PCR was run in triplicate and the average cycle number (Ct) at which amplification rose above the background threshold was determined. The amount of a specific target transcripts was then normalized to the amount of the housekeeping *Actb* transcripts by subtracting the Ct of the target from the Ct of *Actb* (ΔCt). The relative mRNA expression of the target gene was then calculated using a relative quantification method (-2^ΔΔCt^) [48], where ΔΔCt = ΔCt_q_-ΔCt_cb_; q was a target gene expressed in subcongenic mice and cb was the calibrator (the target gene expressed in B6^*znt7*-KO^ mice).

### Data and statistical analysis

Phenotypic results are presented as the mean ± S.E. A two-way ANOVA with repeated measures was used in comparisons between the test groups (Figs. 2, 3, and 4). Student’s *t* test was used in comparisons of the test groups in Fig. 5. Differences were considered to be significant at *p* < 0.05.

## Additional file

Additional file 1: **Table S1.** Primer pairs used in the study. (DOCX 14 kb)

## Abbreviations

129P1: 129P1/ReJ
AUC: Area under the curve
B6: C57BL/6J
IPGTT: Intraperitoneal glucose tolerance test
IPITT: Intraperitoneal insulin tolerance test
KO: Knockout
QTL: Quantitative trait locus
Slc30a7: Solute carrier family 30 member 7
SNP: Single nucleotide polymorphism
Znt7: Zinc transporter 7
