# Supplementary material for: Subcongenic analysis of a quantitative trait locus affecting body weight and glucose metabolism in zinc transporter 7 (znt7)-knockout mice
Source: BMC Genet. 2019 Feb 18;20:19. doi: 10.1186/s12863-019-0715-2 (PMC6378724; doi:10.1186/s12863-019-0715-2)
Supplement: Supplementary file 1 — Table S1. Primer pairs used in the study. (DOCX 14 kb) [file 12863_2019_715_MOESM1_ESM.docx]

| Gene | 5’-forward primer-3’ | 5’-reverse primer-3’ |
| --- | --- | --- |
| 4933405O20Rik | CATTCTAGGGAGGTAGTGTGTGA | CTCCAGGAATCATAGCCACCG |
| Actb | TCATGAAGTGTGACGTTGACATCCGT | CCTAGAAGCATTTGCGGTGCACGATG |
| Ano5 | CACCGCCTGAGAAGCGATT | ACAAAGTCGATCTGCCGGATT |
| Atp10a | CCGCCTAGCCAATGTGTACTT | TGACCGCCAGAATGAAGAGC |
| Chrna7 | AAATCCACCGAGCGATCACC | CTGAGCATGTAACTGTGCAGC |
| Cyfip1 | GAGTCCCTCATTGCAGACAAA | CCCCTCAAGGCTACTTCTCAA |
| E030018B13Rik | CAGAGATGCAGACCGTGGAG | GCTTCTGGTGTGCCCAAATG |
| E2f8 | TTTTCTGAGCCACATAAAAGGGG | CTTCCTTGGGCTTGGTTGGT |
| Fancf | CCTGGGTCCCTACACAGATG | ACGATCCCACACTCAAGCAC |
| Gas2 | CAGACATGAAGCTAATTTGCTGC | GCAAGGCACCATTATCCAACT |
| Herc2 | AGCCAAGTCAATCCTGGAAA | CCAGGGCTTGCTGTTCTTTA |
| Htatip2 | CTGGAGCGGAAGGGTTTGTT | GGAGGACAGCAAGTTGAAATGTT |
| Klf13 | TGCGAGAAAGTTTACGGGAA | TGCGAACTTCTTGTTGCACT |
| Luzp2 | CAGCTCTTGACAGGGAATCAC | CCAAACAGGAACTACCCTCATC |
| Magel2 | ACAAGCATGAATACCCCGAG | ACTGCACAGGCTCCAAAACT |
| Mkrn3 | ACAGGTGTGCATACCCCCA | GCAGGCCCTTCTATGAGCTTC |
| Nav2 | GACTGAGATGCCGAAAAAGTCC | ATTCCTGGTTTTGGTATGCCG |
| Ndn | CTGAGGCTGACCAATCTCCAC | GCCACCCTGTCTAGCTCCT |
| Nell1 | TGCAATAGGATTTATGAGCGTGT | GCCATCTTGGATGATTCCTTTGA |
| Nipa1 | GTGTCCAGCCTAGTGAACGG | CCCACCACACAATGTCTGTTAG |
| Nipa2 | AAAGGATAGCGTTTCCAAGAA | TTTCAATCTTAACTGATAGTCCA |
| Oca2 | ACGGTGCTGGGATTTGTCAT | AGTCGCCCACTCTACTCTGT |
| Otud7a | GTCTCTAGCCTGCTTCCAAAC | GTGGACCGAACAAAGTCTGAC |
| Peg12 | TGAGTGTCTGTGCTTCTGGC | CCCCACTTGAGTAGAGCAGC |
| Prmt3 | CAGAGCACCAAAACACACTGG | TCAGGGTCACAATGAGGGAAC |
| siglech | GGAGGCAAAACATGGAATTTCTG | CACATCACATTGGTAGGACGAC |
| Svip | ACTCGGGGGATTTTGGACATC | CCGATGTCGCCATCTGCTT |
| Snrpn | CAAGGTGGTGGAATTCAAGG | AATAAAGGTTCGATGCAGGC |
| Snurf | GCAACTTCAAGGTGGTGGA | GGTTCGATGCAGGGCTATTA |
| Trpm1 | ATCCGAGTCTCCTACGACACC | CAGTTTGGACTGCATCTCGAA |
| Tubgcp5 | ACACAGGTCATTCGGGAGAC | GCCATACGCTGCTATTTGTTCC |
| Ube3a | GAGGACATTGAAGCTAGCCG | CATTTCCACAGCCCTCAGTT |
| Zdhhc13 | TCGCAGTGCAGGAATCACAG | GGCAGCCCAGTGAAGAAGA |

Table S1. Primer pairs used in the study
